# Review of emergency obstetric care interventions in health facilities in the Upper East Region of Ghana: a questionnaire survey

**DOI:** 10.1186/s12913-018-2980-6

**Published:** 2018-03-15

**Authors:** Minerva Kyei-Onanjiri, Mary Carolan-Olah, John Koku Awoonor-Williams, Terence V. McCann

**Affiliations:** 1 Centre for Chronic Disease, College of Health and Biomedicine, Victoria University, PO Box 14428, Melbourne, VIC 8001 Australia; 2 Ghana Health Service, Accra, Ghana

**Keywords:** Ghana, Emergency obstetric care, Maternal mortality, Obstetric complications, Health facility review, Signal functions

## Abstract

**Background:**

Maternal morbidity and mortality is most prevalent in resource-poor settings such as sub-Saharan Africa and southern Asia. In sub-Saharan Africa, Ghana is one of the countries still facing particular challenges in reducing its maternal morbidity and mortality. Access to emergency obstetric care (EmOC) interventions has been identified as a means of improving maternal health outcomes. Assessing the range of interventions provided in health facilities is, therefore, important in determining capacity to treat obstetric emergencies. The aim of this study was to examine the availability of emergency obstetric care interventions in the Upper East Region of Ghana.

**Methods:**

A cross-sectional survey of 120 health facilities was undertaken. Status of emergency obstetric care was assessed through an interviewer administered questionnaire to directors/in-charge officers of maternity care units in selected facilities. Data were analysed using descriptive statistics.

**Results:**

Eighty per cent of health facilities did not meet the criteria for provision of emergency obstetric care. Comparatively, private health facilities generally provided EmOC interventions less frequently than public health facilities. Other challenges identified include inadequate skill mix of maternity health personnel, poor referral processes, a lack of reliable communication systems and poor emergency transport systems.

**Conclusion:**

Multiple factors combine to limit women’s access to a range of essential maternal health services. The availability of EmOC interventions was found to be low across the region; however, EmOC facilities could be increased by nearly one-third through modest investments in some existing facilities. Also, the key challenges identified in this study can be improved by enhancing pre-existing health system structures such as Community-based Health Planning and Services (CHPS), training more midwifery personnel, strengthening in-service training and implementation of referral audits as part of health service monitoring. Gaps in availability of EmOC interventions, skilled personnel and referral processes must be tackled in order to improve obstetric outcomes.

**Electronic supplementary material:**

The online version of this article (10.1186/s12913-018-2980-6) contains supplementary material, which is available to authorized users.

## Background

Globally, approximately 289,000 women died as a result of pregnancy and childbirth related complications in 2013 [1], and about 5.7 million women experience severe maternal complications annually [2]. Low- and middle-income countries account for 99% of global maternal mortality, with most occurring in sub-Saharan Africa and southern Asia [1]. Similarly, the incidence of severe maternal morbidity is estimated to be highest in low- and middle-income countries [3]. Although estimates vary, for every maternal death, many other women suffer a pregnancy-related illness, sometimes with long-term debilitating consequences [3–5].

Reduction in maternal morbidity and mortality is a priority health sector goal for Ghana’s Ministry of Health, with ongoing efforts including provision of antenatal care and attendance at birth by skilled health personnel [6]. Recent reports indicate that, between 2008 and 2014, 97% of women who gave birth in Ghana received antenatal care from a skilled provider at least once, 73% of births occurred in a health facility and 74% were attended by a skilled provider [7]. Nevertheless, considerable health disparities persist throughout the country, and the maternal mortality rate per 100,000 live births remains high at 380 in 2013, compared with 89 in Algeria and 15 in Libya [1]. Many of these deaths result from inadequate EmOC and poorly equipped facilities [8]. Women’s needs in an obstetric emergency vary considerably; however, key issues include a lack of life-saving interventions in maternity care facilities, a lack of skilled attendants [8], and an absence of standardised referral procedures such as use of referral forms and access to emergency transportation [9]. Assessing health facilities’ capacity to treat obstetric emergencies is an important part of addressing poor maternal health outcomes as such information is vital for health system planning and implementation. This survey, conducted as part of a broader study of EmOC services in the Upper East Region of Ghana, provides baseline information on EmOC services.

In recent years, a number of efforts have been made to reduce barriers to maternity care services in Ghana. For instance, the free maternal healthcare policy was introduced in 2008 as part of the National Health Insurance Scheme, which entitles pregnant women to six free antenatal care visits, birthing care, two postnatal care visits and newborn care for up to three months [10]. Additionally, a type of primary health service (CHPS), was commenced in 1999. The main purpose of CHPS was to increase access to primary health services by reducing the distance to health facilities in communities [11], especially in remote and underserved communities [6]. However, although increased access is important, it may not necessarily improve birthing outcomes if services are not matched to client needs. This may be the case with CHPS services which are mostly manned by auxiliary nurses called community health nurses, with two years training in the provision of basic health services for minor ailments [12]. Generally, CHPS facilities do not routinely provide birthing services, particularly if there is no midwife assigned; although antenatal, postnatal and family planning services are often offered. Some community health nurses in CHPS facilities have undergone short in-service training programmes that equip them to provide emergency, non-complicated birthing services; nonetheless, not all those trained offer such services. This dilemma has arisen due to a shortage of midwifery personnel and the advanced age of many practising midwives in Ghana. As a result, it has become necessary to adopt measures, such as task shifting and training different categories of midwives to ensure continued access to skilled birthing services. Presently, community health nurses can opt to receive a two-year midwifery training which prepares them to provide basic obstetric care [12]. Furthermore, the Ghana Health Service is implementing a policy shift of discouraging the use of traditional birth attendants (TBAs) for birthing services [13].

In addition to measures mentioned, such as efficient health financing, provision of health infrastructure and personnel, the responsiveness of a health system to clients’ needs requires monitoring. One way to measure the availability or effectiveness of maternal health services is to examine their capacity to respond to obstetric emergencies or provide EmOC interventions. The term *signal functions* is often used to denote essential or key interventions for the treatment of direct obstetric complications which account for most maternal deaths [14]. The components of basic and comprehensive EmOC interventions are based on evidence that the majority of maternal deaths worldwide are due to five direct causes; namely, severe bleeding, unsafe abortion, hypertensive disorders, obstructed labour and sepsis. These causes can be treated effectively in well-staffed, well-equipped health facilities [2]. The *Monitoring Emergency Obstetric Care: a Handbook* [14], provides an organising framework for assessing evidence-based clinical interventions that an EmOC facility offers, specifically, components of basic and comprehensive EmOC capabilities (Table 1). The selected components are considered necessary to protect women against preventable deaths and should not be viewed as a comprehensive list for service provision at any level of care.

**Table 1:** Composition of basic and comprehensive EmOC (signal functions)

Basic EmOC	Comprehensive EmOC
a) Administer parenteral antibiotics b) Administer uterotonic drugs (i.e., parenteral oxytocin) c) Administer parenteral anti-convulsants for pre-eclampsia and eclampsia d) Perform manual removal of placenta e) Perform removal of retained products (e.g., manual vacuum aspiration) f) Perform assisted vaginal delivery (forceps, vacuum extraction)	- All 6 basic functions plus: g) Perform blood transfusion h) Perform surgery (e.g., caesarean section)

Adapted from World Health Organization, UNFPA, UNICEF and Averting Maternal Death and Disability (2009)

Assessment of facility-based obstetric interventions is based on the combination of basic and comprehensive obstetric services provided by a health facility. If a facility provides services a-h, it is categorised as providing comprehensive EmOC; a-f indicates basic EmOC; and any discrepancy in a-f is rated as not providing basic EmOC.

Another measure, which is useful for collecting data on the status of health services, is the Service Provision Assessment developed by The DHS Program [15]. The Ghana Service Provision Assessment, conducted in 2002, is very broad and includes a segment on maternity care (Section 2). The present study drew on Section 2, *Maternal Health Services*, which explores the availability of basic and comprehensive EmOC and referral services for mothers. Selected questions from the sub-section, *Part 2: Facility Inventory Questionnaire,* were particularly useful for this study [16].

The aim of this study was to assess the status of EmOC facilities. We focused particularly on the provision of life-saving obstetric interventions, availability of skilled personnel, service hours, communication and transport for emergency referrals.

## Methods

### Study setting

The Upper East Region is located in the north-eastern part of Ghana with a population of over one million. The region has a predominantly rural population of 79% [17] and is the third poorest region with about 44% of the population classified as poor [18]. The region currently has 13 administrative districts/municipals. Health service delivery is organised at four levels of care in a total of 267 facilities, comprising about 159 primary health-care units (CHPS facilities), 101 health centres and clinics, six district hospitals and one regional hospital, which is the major referral facility. Only a few private health facilities operate in the region [9].

### Study design

A cross-sectional survey was conducted across the four levels of care. Status of EmOC was assessed through an interviewer administered questionnaire to directors/in-charge officers of maternity care units in selected facilities (Additional file 1). The questionnaire was developed from two documents (described above), *Monitoring Emergency Obstetric Care: a Handbook* [14], and Appendix C of the *2002 Ghana Service Provision Assessment* [16]*.* It contained 26 closed questions on the nature and types of services provided. Levels of EmOC availability were defined as basic or comprehensive as shown in Table 1.

### Sample size

Not all 267 health facilities in the region provide birthing services. The main inclusion criterion for the survey was healthcare facilities that provided birthing services in the region. Based on available information, the number of facilities that met this inclusion criterion was 160, making it the study population. The sample size calculation was therefore based on this estimate of 160 health facilities (*N* = 160). The sample size was calculated using the formula n = [(z^2^*p*q) + ME^2^]/[ME^2^ + z^2^*p*q/N] at a confidence level of 95%, (standard value of 1.96), margin of error (ME) of 5% (standard value of 0.05), and population proportion (**p)** ***of*** .50. This calculation yielded a minimum sample size of 114 health facilities. Although the minimum sample arrived at was 114, the facilities were oversampled and 123 facilities were approached for data collection.

### Data collection

Prior to data collection, contact details of directors/in-charge officers of selected facilities were obtained from the district health directorates. Prospective participants were then contacted by telephone, offered information about the nature and purpose of the study and given time to consider it. Information to participants’ forms were provided and participation was voluntary. Interviews were conducted at the health facilities at a prearranged time with the directors/in-charges. Interviews lasted between 30 and 35 min.

### Data analysis

Data were entered into Epi Info™ 7 (Version 7.1.5) and subsequently exported to IBM® SPSS® Statistics Version 22.0 for analysis. Data were analysed using descriptive statistics and results shown mainly as percentages and frequency distributions.

## Results

Overall, of 123 healthcare facilities approached, 120 consented to participate in the survey. Reasons for declining included too busy to participate (*n* = 2) and personal reasons (*n* = 1). The 120 participating facilities included 17 clinics, 52 CHPS centres, 41 health centres, 9 hospitals (including private hospitals) and 1 maternity home. All 13 districts/municipals in the region were represented in the study. Public health facilities comprised about 91% of the total sample, which is consistent with birthing service use.

### Provision of emergency obstetric interventions

Eighty per cent (*n* = 96) of the health facilities did not meet the criteria for provision of basic or comprehensive EmOC. Only 7.5% (n = 9) of facilities provided comprehensive EmOC and the remaining 12.5% (*n* = 15) provided basic EmOC. Of the 15 facilities providing basic EmOC, 13% (*n* = 2) were private. Of the 96 facilities that did not offer EmOC, 95% (*n* = 91) were public healthcare facilities. Public health facilities represented approximately 55% (*n* = 5) of facilities offering comprehensive EmOC. Approximately 10% (*n* = 12) of facilities almost met the basic EmOC criteria as they offered all but one basic EmOC intervention, and another 21% (*n* = 25) offered all but two.

Out of 15 basic EmOC facilities, the majority (74%) were clinics/health centres and 20% were CHPS facilities. Most facilities that met the criteria for comprehensive EmOC were hospitals (67%) and the rest were clinics. More than half of the health facilities categorised as not providing EmOC (51%) were CHPS facilities and approximately 46% were health centres/clinics. Considering the majority of facilities in the study were at the lowest or middle levels of referral networks, these results are consistent with the study setting, where each level of care offers a specific range of maternity services, which is further determined by existing categories of staff and their particular skills set as well as available equipment and supplies.

Of the key obstetric interventions outlined in Table 2, the most commonly performed basic EmOC service was assisted vaginal delivery, followed by administration of parenteral oxytocics. Overall, caesarean section was the least offered comprehensive EmOC procedure, closely followed by blood transfusion. The provision of parenteral anticonvulsants and manual removal of placenta were the least commonly performed basic EmOC interventions.

**Table 2:** EmOC interventions provided across all facilities

	EmOC Interventions	Number	Health Facilities (%)
Basic EmOC	Parenteral antibiotics	80	66.7
	Parenteral oxytocics	90	75.6
	Parenteral anticonvulsants	38	31.7
	Manual removal of placenta	57	47.5
	Removal of retained products of conception	58	48.3
	Assisted vaginal delivery	96	80.0
Comprehensive EmOC (including all of the above)	Blood transfusion	14	11.8
	Caesarean section	12	10.0

**Fig. 1:**
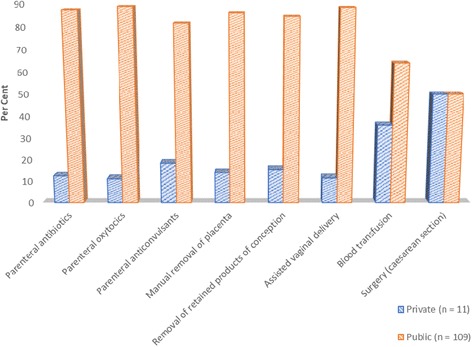
Private and public health facilities’ coverage of EmOC interventions

Other than the provision of caesarean section, overall coverage of EmOC interventions in public health facilities were high, ranging from 64% to 89%, compared to private facilities, which ranged from 11% to 36% (Fig. 1). Additionally, availability of EmOC facilities differed between districts. Nine out of 13 districts/municipalities had at least one basic EmOC facility while seven out of 13 had at least one comprehensive EmOC facility.

Approximately 13% of facilities reported that they facilitated home births routinely; the majority (77.5%) offered the service only in emergency situations while 10% did not offer this service. Additionally, just 38% of the total sample reported providing post abortion care in their facilities.

### Availability of skilled personnel and service hours

Most health facilities (91%) reported having a trained health provider (not necessarily a person with midwifery skills) present at all times, and most (86%) provided maternity care services 7 days a week. Seventy-three per cent of facilities had a midwife or doctor present or on-call at all times, whilst 14% had trained health personnel present who was not a midwife or a medical doctor. About 8% of facilities did not have a person with midwifery skills present at all times (Table 3). Thirty-five per cent of respondents indicated that the skill mix of clinical staff in their units was not appropriate for the types of caseloads they received, and 70% reported that their maternity units were not well-equipped for the services they needed to provide.

**Table 3:** Availability of persons with midwifery skills at facilities

Type of staff present	Number	%
Personnel without midwifery skills present	10	8.3
Trained Health provider (not a midwife or doctor) - may be a community health nurse or community health officer	17	14.2
Midwife or doctor present or on call	88	73.3
Other personnel on call (not a midwife) such as those trained on-the-job.	1	0.8
Other personnel present (not a midwife)	4	3.3
Total	120	100.0

### Referral processes, communication and emergency transport

Most health facilities (94%) had a standardised or printed referral form for obstetric referrals. Eighty-three per cent reported having a standard procedure for transferring maternity patients to other facilities. Sixty-four per cent (*n* = 75) of facilities had a working telephone or shortwave radio for communication while the remainder had none. Of those without a working telephone, over half (56%) could not reach one within five minutes of the facility.

Thirty-four per cent of facilities had an emergency vehicle for transporting referred clients to other facilities; however, 40% indicated that they called another facility (24 h a day) to send their emergency vehicle for referrals. The most common means of transportation between facilities was by a car (52%), followed by an ambulance (21%) and a motorised tricycle (10%).

One hundred and four facilities responded to the question about travel time. The mean travel time to the nearest referral facility was 37 min with a range of 5 to 120 min using the various means of transport available to them.

## Discussion

We examined provision of life-saving obstetric interventions in health facilities in the Upper East Region of Ghana, one of the poorest regions in the country [18]. Findings emerged regarding a range of issues, including low availability of basic and comprehensive EmOC facilities, inadequate numbers of skilled personnel and challenges in referral processes.

### Availability of basic and comprehensive EmOC facilities

Overall, the results show low availability of EmOC interventions across the region, which is consistent with findings of an earlier study [19] in a more affluent region of Ghana. This situation limits EmOC access for the populations concerned. Methods of rating EmOC compliant services may contribute somewhat to the low availability of EmOC services and there may be reasons for not providing those services in the period under study. For instance, Paxton et al. [20] point out that some facilities may not receive enough cases of obstetric complications to perform all emergency obstetric interventions within a three-month reference period.

In implementing changes, it is practical and cost-effective to initially identify facilities that almost meet the minimal standards as these will likely require the least investment for quality improvement. The dispersed rural settlements found in most parts of the Upper East Region means that the CHPS model of primary healthcare in Ghana has the potential to reach a greater number of maternal healthcare users, if up-scaled properly. In addition, as the Ghana Health Service continues its policy shift of investing less in training and use of TBAs in maternity care services [13] in a bid to discourage their use, it is necessary that all CHPS facilities are upgraded to at least a basic EmOC status. Although current efforts to train more midwives will significantly advance obstetric care [12], the time lag between phasing out of TBAs and increased access to efficient EmOC will likely result in continued dependence on TBAs, particularly, in rural settings. As access to EmOC improves, maternal morbidity and direct obstetric deaths can be reduced significantly with appropriate interventions [2].

### Availability of skilled personnel

Availability of skilled personnel directly affects maternal and infant outcomes in low-resource settings [2]. In the absence of trained health personnel, competent in the delivery of obstetric services, reducing preventable deaths is challenging [2]. Our study indicated that provision of parenteral anticonvulsants and manual removal of placenta were the least commonly performed basic EmOC interventions. This trend could be a reflection of a combination of factors, including absence of personnel qualified to provide those services and non-availability of needed equipment and supplies. Similarly, the limited provision of post-abortion care in the region may be partly attributable to poorly equipped facilities and a poor health personnel skill-mix. In addition, individual beliefs and socio-cultural factors may play a significant role. A study of attitudes of physicians toward establishing safe abortion units in Ghana showed that only 45% expressed a willingness to conduct pregnancy terminations themselves and 36% expressed a willingness to participate in counselling only [21]. Finally, based on gap analysis data from the region’s human resource unit, there is a need for approximately 500 more midwifery personnel to meet the maternal health needs of women, with about two-thirds of this number classified as short-term or priority need (Human resource officer, Upper East regional health directorate, personal communication, May 7, 2015) which represents a major shortage. Challenges in the recruitment and retention of healthcare personnel, especially in rural areas, contribute to maldistribution of midwifery workforce to the disadvantage of rural dwellers. Lack of opportunities for further education, poor working conditions, poor quality of education for children and lack of social amenities have been cited as reasons which influence midwifery personnel’s willingness to work in rural areas [22].

### Referral processes

Key components of any referral process include use of referral forms, communication with receiving facilities, health workers accompanying referred clients, and access to emergency transportation especially in remote areas with poor road and transportation networks [9]. Almost one-fifth of facilities had no standard procedure for transporting clients. This significant deficit could be met by ensuring adherence to standardised referral guidelines by frontline healthcare workers. Health personnel may be unable to fully facilitate referral processes due to a lack of access to telephones for communication as found in this study. Inefficient referral systems are often associated with delays or non-compliance to referrals, reliance on alternative care such as TBAs and herbalists, which leads to a worsening of complications [23], and maternal deaths [23–25]. In an intervention study in Uganda, reliable communication and transport services increased access to hospital deliveries increased by over 50% per year [26]. Another study [9] has demonstrated that use of referral audits that are built around a problem-solving approach can significantly increase adherence to standardised referral guidelines, which has implications for reducing maternal morbidity and mortality. Furthermore, the timeframes for travel found in this current study may be too long for women to reach and obtain the EmOC they require, and consequently effect the outcomes of referred clients. Poor transportation systems have consequences, especially for poor rural women and their families, who are less likely to comply with referral instructions due to financial difficulties associated with arranging their own transportation [27, 28]. Additionally, such challenges may plunge poor households further into poverty [29] and generally discourage health service users from seeking skilled care in the future. In settings where resources for comprehensive EmOC are unavailable, transport and communication networks need to be improved to increase access to care.

### Strengths and limitations

A major strength of this study was the large and representative sample obtained in the study area, which was possible as responses were de-identified to ensure confidentiality. However, some limitations were identified. As this study was reliant on self-report by respondents, there is a possibility of bias resulting from under-reporting. To help counter the possibility of such bias, anonymity and confidentiality of responses was assured. In addition, the results cannot be generalised to the whole country, as the circumstances and context of health service provision may differ in other regions. Nevertheless, this study has important policy implications that are relevant to obstetric care in Ghana, especially in regions with predominantly rural and relatively high levels of impoverished populations.

### Implications for practice

This study brings to light the potential of modest improvements in health facility capacities which may increase the provision of EmOC in the region, particularly for key services with very low coverage, such as post-abortion care. Ghana can also strengthen the uniquely placed community-focused CHPS system to increase utilisation of maternal health services. Training, equitable distribution, and retention of skilled personnel, must be a consideration for future planning to improve the skill-mix in maternity units. However, without an adequate supply of equipment, readily accessible transport and adequate communication systems, skilled personnel will be unable to provide required assistance to their clients. Given the importance of referral procedures and its interconnection with receipt of further treatment in obstetric emergency situations, the role of referral audits and in-service training of maternal health personnel, in appropriate and timely referral processes cannot be overemphasised, in efforts to improve birthing outcomes.

## Conclusion

Access to EmOC for women in the Upper East Region is limited by multiple factors, including inadequate equipment and personnel, communication and transport problems as well as poor referral practices. We recommend that, as on-going changes are made, health facility reviews should be conducted regularly for up-to-date data on the status of obstetric services to guide policy directions in maternity care and support further improvements.

## Additional file

Additional file 1: Facility review questionnaire. Description of data: Instrument for data collection (DOC 94 kb)

## Abbreviations

CHPS: Community-based Health Planning and Services
EmOC: Emergency obstetric care
TBAs: Traditional Birth Attendants
