# Supplementary material for: Review of emergency obstetric care interventions in health facilities in the Upper East Region of Ghana: a questionnaire survey
Source: BMC Health Serv Res. 2018 Mar 15;18:184. doi: 10.1186/s12913-018-2980-6 (PMC5855967; doi:10.1186/s12913-018-2980-6)
Supplement: Supplementary file 1 — Facility review questionnaire. Description of data: Instrument for data collection (DOC 94 kb) [file 12913_2018_2980_MOESM1_ESM.doc]

**FACILITY REVIEW QUESTIONNAIRE**

**Box A: Facility's Possible EOC Status**

To be done at **district** level before completion of this form.

***Tick ONE***

**Comprehensive EOC __**

**Basic EOC ___**

**Box B: Facility's Actual EOC Status**

To be done at **facility** level after completion of this form.

***Tick ONE***

**Comprehensive EOC __**

**Basic EOC ___**

1. Location of facility:
2. Type of locality:

3. Type of facility: a) Hospital ___ b) Maternity home ___ c) Health Centre ___

d) Clinic --- e) Community-Based Health Planning and Services ___

f) Traditional Birth Attendant ___ g) other (specify) _______________________

|  |  |  |  |  |  |  | **Box: Determination** | |  |
| --- | --- | --- | --- | --- | --- | --- | --- | --- | --- |
| *Check Yes or No for each of the following items (a-h)* | |  |  |  |  |  | **of EOC status** | |  |
|  |  |  |  |  |  |  | *(Use Q5. Check only ONE.)* | |  |
| **5. Were the following services** |  |  |  |  |  |  |  |  |  |
|  |  |  |  |  |  |  |  |  |
| **performed at least once during the last 3 months?** | Yes | No |  |  | ! If **ALL** of 5a–h = Yes, check: | | |  |  |
|  |  |  |  |  |  |  |  |  |  |
| (a) Parenteral antibiotics |  |  |  |  |  |  | COMPREHENSIVE EOC |  |  |
| (b) Parenteral oxytocics |  |  |  |  |  |  |  |  |  |
| (c) Parenteral sedatives/anticonvulsants |  |  |  |  | ! If **ALL** of 5a–f = Yes **AND** | | |  |  |
| (d) Manual removal of placenta |  |  |  |  |  | 5g **OR** 5h = No, check: | |  |  |
| (e) Removal of retained products |  |  |  |  |  |  | BASIC EOC |  |  |
| (f) Assisted vaginal delivery |  |  |  |  |  |  |  |  |  |
| (g) Blood transfusion |  |  |  |  | ! If **ANY** of 5a–f = No, check: | | |  |  |
| (h) Caesarean section |  |  |  |  |  |  | **NOT** EOC |  |  |
|  |  |  |  |  |  |  |  |  |  |

4. Facility ownership: a) Public ___ b) Private _

1. ***Does this facility offer normal maternity care services?***

Yes........................................... 1

No............................................. 2

1. ***Is there a trained health provider present at the facility at all times (24 hours/day)?***

Yes, trained provider always present ................1

No ……………….............................................2 If yes, skip to 9

1. ***Is there a trained health provider available on call at all times after hours?***

Yes, ……………………. 1

No..................................... 2

1. ***Routinely, how many days each week is this facility/unit open for maternity care services?***

Number of days……………..

1. ***Is a person with midwifery skills present at the facility or on call 24 hours a day, including weekends, to provide delivery care?*** *(If Midwife (MW) or Doctor (MD) not ALWAYS presents or on-call, but someone with midwifery skills is, circle the NON-MIDWIFE responses).*
2. Non MW or MD present ........... 1
3. Yes MW or MD on call.............. 2
4. Yes, non-midwife present………3
5. Yes, non-midwife on call …….. .4
6. No ............................................... 5
7. ***Does this facility have a health personnel who can perform a caesarean section present in the facility or on-call 24-hours a day (including weekends)***?

Yes, present ........................... 1

Yes, on call..............................2

No.............................................3

1. ***Does this facility currently provide post-abortion care?***

Yes...........................................1

No.............................................2

1. ***Do midwives/care providers routinely provide home birth assistance or attend home birth emergencies as a part of the facility’s services?***

Yes, routinely............................1

Yes, emergency only................ 2

No............................................. 3

1. ***Does the facility have a system for routinely reviewing maternal deaths or "near-miss deaths"?***

Yes, ……………................1

No……………………........2

1. ***What is the most commonly used means by which women are transported from home to this facility during obstetric emergencies?***

Carried by people................... 1

Animal drawn vehicle …......2

Motor vehicle........................3

Bicycle .................................4

Combination of above...........5

Other (specify)____________6

Don’t know .......................... 7

1. ***Does this facility have a procedure for transporting women to another facility (if necessary), in an obstetric emergency?*** *Record “not applicable” if facility is the referral (receiving) facility.*

Yes ...........................................1

No............................................ 2

Not applicable...........................9

1. ***Is a printed referral form sent with referrals from this facility?*** *(If the facility is a referral (receiving) facility, then circle “4” for referral facility).*

Yes ................................................ 1

No form, use letter head ................. 2

No................................................... 3

Referral facility .............................. 4

Don’t know .................................... 8

| 24 hours | Normal  Facility hours (< 24 hours) | No set times | Not used |
| --- | --- | --- | --- |
| 1 | 2 | 3 | 8 |
| 1 | 2 | 3 | 8 |
| 1 | 2 | 3 | 8 |
| 1 | 2 | 3 | 8 |

1. ***Circle for each of the following, the most commonly available means used by this facility for emergency transportation:***
2. Emergency vehicle onsite at facility
3. Multi-use vehicle available at facility. May be used for emergencies
4. Call other facility to send emergency vehicle
5. Rental/hire vehicle arrangement when needed (with some financial support from facility)
6. ***In recent times, how long has the vehicle been operational?***

Vehicle not operational………………………….1

Under one month………………………………..2

One to three months……………………………..3

Three to six months……………………………...4

Six to nine months……………………………….5

Nine months to one year…………………………6

One year and over………………………………..7

1. ***What is the most commonly used means for transporting pregnant women from this facility to the nearest referral facility during an obstetric emergency?***

Carried by people ............ 1

Animal drawn vehicle........2

Motor vehicle ....................3

Combination of above .......4

Other (specify) _________________5

Don’t know.........................6

1. ***How long does it take, using this form of transportation, to get to the nearest referral***

***facility?*** *(Note: if call elsewhere to obtain vehicle, record average time from call to patient arrival at referral facility)*

Minutes___________________

Don’t know..............................9

1. ***Does this facility have a working phone or short-wave radio?***

Yes ..................................1 If yes, go to 24

No................................... 2

1. ***Is there a phone or short-wave radio within five minutes distance from the facility that staff can use in an emergency? IF YES: Is that phone or short-wave radio available 24 hours a day?***

Yes, available 24 hours.............................................1

Yes, not available 24 hours ......................................2

No, none within 5 minutes........................................3

1. ***In your opinion, is the skill mix in this facility appropriate to cope with the patient flow and case mix received?***

Yes ..................................1

No................................... 2

1. ***In your opinion, are the maternity wards adequately equipped to offer designated maternity care services?***

Yes ..................................1

No................................... 2

1. ***Is the operating theatre (if any) in good repair and fully equipped with drugs and surgical equipment to perform life-saving procedures, when required?***

Yes ..................................1

No................................... 2

None available………….3
